# Fracture Resistance of Laboratory Composite Versus All-Ceramic Restorations in Class II Inlay Cavity Preparations: An In Vitro Study

**DOI:** 10.7759/cureus.44711

**Published:** 2023-09-05

**Authors:** Shreya Agarwal, Vineet Gupta, Srinkhala, Shreya Singh, Poorvi Saxena, Jaydip Marvaniya

**Affiliations:** 1 Department of Conservative Dentistry and Endodontics, Awadh Dental College and Hospital, Jameshdpur, IND; 2 Department of Conservative Dentistry and Endodontics, King George's Medical University, Lucknow, IND; 3 Department of Conservative Dentistry and Endodontics, The Dental Home Clinic, New Delhi, IND; 4 Department of Conservative Dentistry and Endodontics, 32 Smiles Multispeciality Dental Clinic, Bangalore, IND; 5 Department of Conservative Dentistry and Endodontics, Geetanjali Dental and Research Institute, Udaipur, IND; 6 Department of Conservative Dentistry and Endodontics, Career Dental College, Lucknow, IND

**Keywords:** inlay cavity preparations, class ii, all-ceramic restorations, lab composite, fracture resistance

## Abstract

Background: A posterior tooth's occlusal surfaces and the proximal surface can be restored by using an inlay, which is an intra-crown cast reconstruction without affecting the cusps of the tooth. When an inlay is prepared using an indirect approach, issues with traditional filling approaches, including poor morphology of the occlusal aspect or proximal aspect, inadequate resistance to wear, or subpar mechanical qualities of the directly inserted filler substance, are overcome.

Aim: The current study was conducted in order to compare and assess the resistance to fracture of dental materials used in the preparation of inlay restorations indirectly, like composite restorations prepared by laboratories indirectly, inlays formed indirectly of monolithic translucent ceramic derived from zirconia, and inlays formed indirectly of traditional monolithic ceramic derived from zirconia.

Methods and materials: For the investigation, 100 human premolars of the maxilla that were extracted recently were chosen. A self-polymerizing acrylic resin was used to incorporate the tooth roots in a band made up of polyvinyl chloride up to 2 mm below the cement-enamel junction. The dimension of the band was 1.3 cm by 1.9 cm. Five categories of 20 specimens of such teeth were formed. Category one, featuring teeth in good condition, acted as the positive control category. The remaining four categories of teeth received inlay tooth preparation. The research samples underwent thermocycling after having been preserved for a full week following the cementation of inlay replacements. Then, in a universal testing apparatus, every sample endured axial compressive force with a metal globe delivered vertically at a crosshead rate of 1 mm/minute. The amount of force necessary to cause a fracture was measured in Newtons (N).

Results:The mean values of resistance against fracture in specimens in categories 1-5 were 1208.87 N, 614.89 N, 733.05 N, 1179.14 N, and 1148.49 N, respectively. The values of fracture resistance in specimens where an inlay cavity preparation was done but not filled were lower than those in traditional monolithic ceramic derived from zirconia and tooth specimens with inlays formed of monolithic translucent ceramic derived from zirconia, and the difference was significant statistically (p=0.001). The values of fracture resistance in composite inlay restorations prepared by laboratories were indirectly lower than those of monolithic ceramic derived from zirconia and tooth specimens with inlays formed of monolithic translucent ceramic derived from zirconia, and the difference was significant statistically (p=0.004).

Conclusion: Within the constraints of the current investigation, we can state that indirect zirconia-based ceramic products offer adequate fracture resistance, but additional research is needed to determine how well these materials hold up under different types of pressures before employing them in clinical tooth restoration.

## Introduction

A posterior tooth's occlusal surfaces and the proximal surface can be restored by using an inlay, which is an intra-crown cast reconstruction without affecting the cusps of the tooth. When an inlay is prepared using an indirect approach, issues with traditional filling approaches, including poor morphology of the occlusal aspect or proximal aspect, inadequate resistance to wear, or subpar mechanical qualities of the directly inserted filler substance, are overcome [1,2]. The material used in this investigation was a nanocomposite called GC Solare Sculpt-Universal Restorative Dental Composite (GC Corporation, Tokyo, Japan). This composite comprises advanced and recent light dispersion technology. They are universally sculptable and feature uniformly distributed single-spread nanofillers that offer outstanding flexibility and durability against wear [3,4].

The requirement for restoration of teeth in the posterior region is linked to the remaining dental structure's biomechanical properties, biocompatibility properties, as well as aesthetic considerations. Zirconia is one of the materials that is frequently used as tooth-colored indirect inlay restorations in teeth in the posterior region. Particularly in terms of utility, zirconia possesses certain qualities that include better aesthetics, good physical strength, and increased mechanical strength [5,6]. The varied properties of each dental material, both functionally and aesthetically, must be taken into consideration while choosing restoration materials for indirect inlay preparations for posterior teeth in order to choose the most appropriate material [7,8].

A lack of knowledge was found in the literature concerning the impact of composite restorations prepared in laboratories and inlays formed indirectly from ceramic derived from zirconia on teeth's ability to withstand fracturing [9,10]. The current study was conducted in order to compare and assess the resistance to fracture of dental materials used in the preparation of inlay restorations indirectly, like composite restorations prepared by laboratories indirectly, inlays formed indirectly of monolithic translucent ceramic derived from zirconia, and inlays formed indirectly of traditional monolithic ceramic derived from zirconia.

## Materials and methods

For the investigation, 100 human premolars of the maxilla that were extracted recently were chosen. The study was conducted at Awadh Dental College and Hospital, Jamshedpur, India. The Ethics Committee of Awadh Dental College approved the study (approval number: IEC/ADC/2022/246). A self-polymerizing acrylic resin was used to incorporate the tooth roots in a band made up of polyvinyl chloride up to 2 mm below the cement-enamel junction. The dimension of the band was 1.3 cm by 1.9 cm. Five categories of 20 specimens of such teeth were formed. Category one, featuring teeth in good condition, acted as the positive control category. The remaining four categories of teeth received inlay tooth preparation.

**Figure 1:**
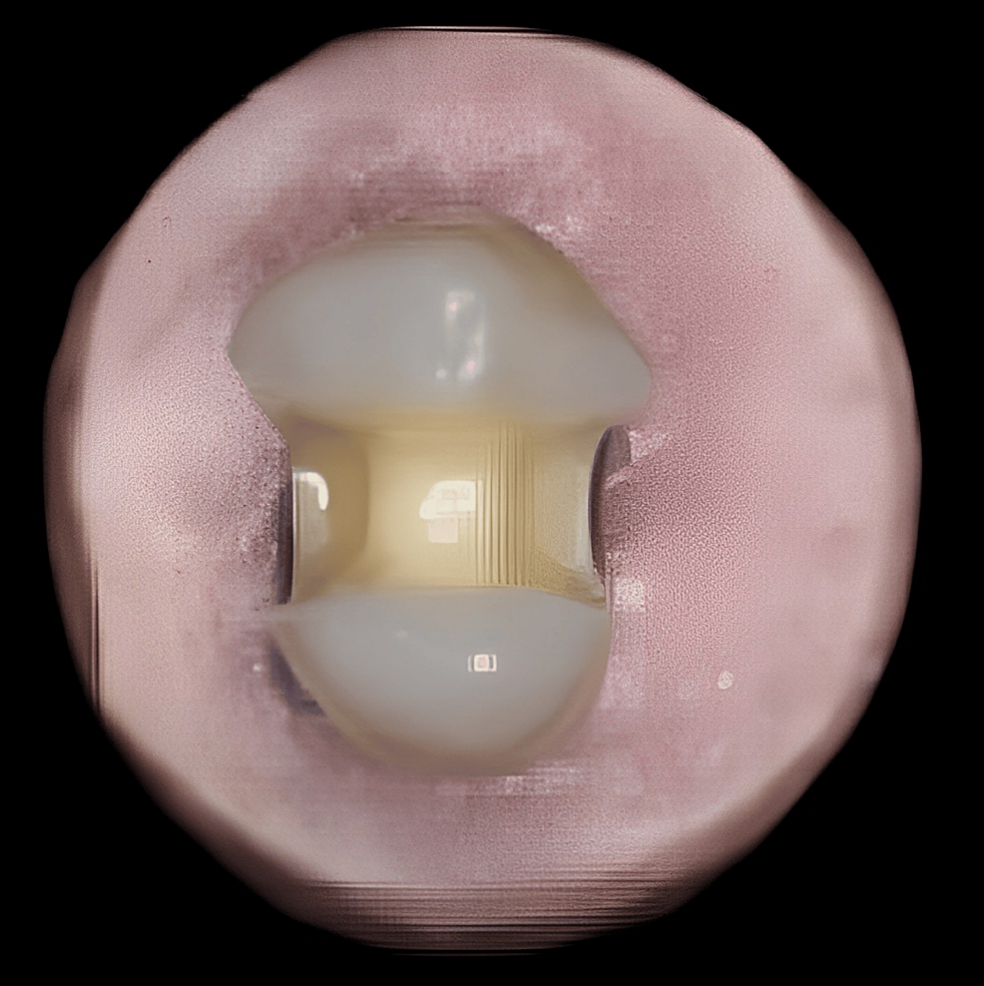
Cavity preparation

Each sample had a standardized inlay cavity constructed for it. After four consecutive preparations, the No. 271 bur was changed to ensure cutting effectiveness. Procedures for the preparation of cavities in each category followed standards for size as given in Table 1. This was accomplished by using a standardized Williams probe to measure the depth of the floor and the width of the isthmus at the occlusal surface. Vinyl-polysiloxane impression-making material was used to obtain prosthodontic impressions of the tooth specimens in which the inlay cavities were prepared. To create a die framework, the impressions were filled with Type IV gypsum. All inlay restorations were created in accordance with the guidelines provided by the manufacturers of the relevant materials (Table 1, Figure 1).

**Table 1:** Categorization of inlay restoration

Category	Number of specimens	Description
Category 1: Positive control category	n = 20	A specimen from this category didn't get any cavity preparations or restoration.
Category 2: negative control group	n = 20	For every specimen in this category, an inlay cavity preparation was done but not filled.
Category 3: Composite restoration prepared by a laboratory indirectly	n = 20	A composite restoration prepared by the laboratory indirectly was used to restore every inlay cavity preparation.
Category 4: Inlay formed of traditional monolithic ceramic derived from zirconia	n = 20	Inlays formed of traditional monolithic ceramic derived from zirconia were used to restore every inlay cavity prepared.
Category 5: Inlay formed of monolithic translucent ceramic derived from zirconia	n = 20	An inlay formed of monolithic translucent ceramic derived from zirconia was used to restore every cavity preparation.

In order to prepare an indirect inlay restoration with composite, there was compression of the composite in layers. Each layer was cured under light for 40 seconds. These procedures were carried out on a prototype die. After forming the occlusal framework, the inlay was heated and cured for 15 minutes at 100°C in the oven. With delicate diamond tips and carbide burs operating at low speeds and gentle pressure, each inlay was meticulously finalized.

To achieve an optical picture, an antireflection varnish was sprayed onto every single die prototype. A completely computerized optical strip-light scanning device was used to scan the model, which was linked to a computer-aided design/computer-aided manufacturing (CAD/CAM) workstation. A specialized version of software designed for inlays was used to generate the framework after accurately marking all the margins of the cavity on the digital photograph. The physical prototypes, after being designed, were machined using VITA In-Ceram® YZ Cubes (VITA Zahnfabrik H. Rauter GmbH & Co. KG, Bad Säckingen, Germany). After that, a carbide bur was used to finalize the milled prototype. Following the drying step, all of the structures were put on a firing rack and then sintered for roughly 7.5 hours at 1530°C, comprising the phase of cooling at 200°C, in a sintering kiln. In order to finish the glaze firing period, all restorations were subsequently heated in a furnace with a vacuum at 700°C and subsequently glazed at 500°C.

**Figure 2:**
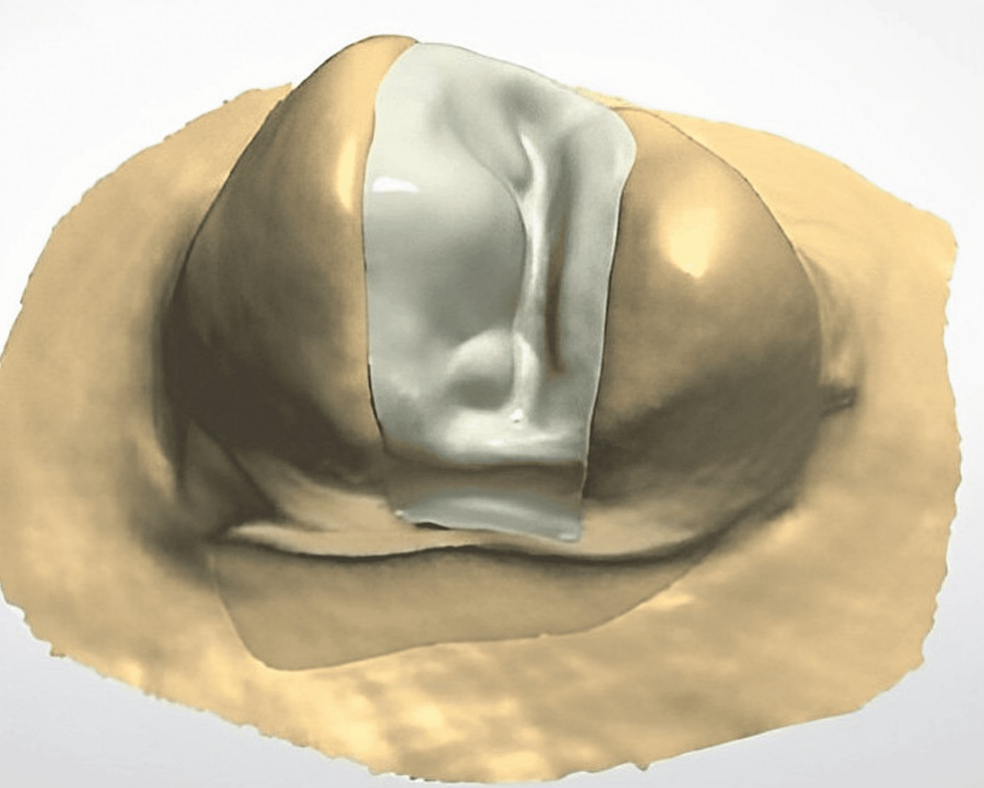
Computer-aided design/computer-aided manufacturing (CAD/CAM) designing

The preliminary frameworks were machined from Cercon XT cubes (Dentsply Sirona, Charlotte, North Carolina, United States) using machining burs within a CAD/CAM machining center (Dentsply Sirona) after similarly preparing inlay patterns, as described in category four. All of the preliminary frameworks were subsequently put on a firing rack, and then sintering was carried out at 1500°C for 4.5 hours, also incorporating a cooling period at 200°C following drying. All ceramic restorations were then finished using a sandblasting procedure with Al2O3 after being heated in a vacuum kiln at 920°C and glossed at 45°C (Figure 2).

**Figure 3:**
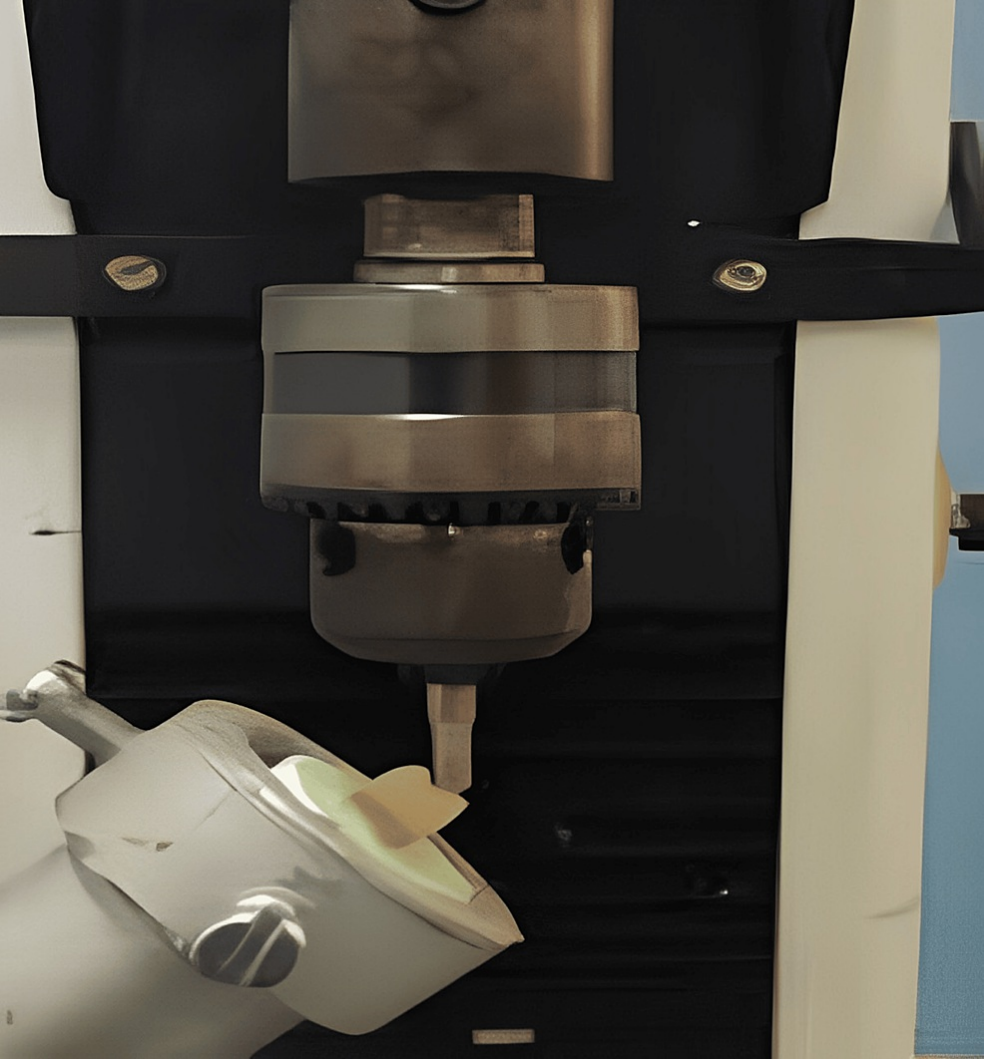
Fracture resistance

Inlay restorations were inserted and fixed using dual-cure adhesive cements following the try-in processes. The research samples underwent thermocycling. All specimens were thermo cycled for 500 cycles from 5-55°C with 30 seconds dwell time and 20 seconds transfer time using a thermo-cycling machine after having been preserved for a full week following the cementation of inlay replacements. Then, in a universal testing apparatus, every sample endured axial compressive force with a metal globe delivered vertically at a crosshead rate of 1 mm/minute (Figure 3). The amount of force necessary to cause a fracture was measured in Newtons (N).

The four categories used to categorize fractured specimens observed under stereomicroscopy (Leica Microsystems GmbH, Wetzlar, Germany) are shown in Table 2.

**Table 2:** Categorization of fractured specimens observed under stereomicroscopy

Categorization of fractured specimens
CS: Cohesive breakage of the experimental tooth
AD: Adherent breakage at the interface of tooth and restoration
CM: Cohesive collapse of the dental restoration's material
CO: Entire breakage of the specimen

The data obtained were tabulated and put to statistical analysis. The category heterogeneity was examined using a two-way analysis of variance (ANOVA) statistical analysis. The significance threshold was established at p<0.05. To determine the degree of significance of the differences among each category, post hoc Bonferroni test was used.

## Results

The mean values of resistance against fracture in specimens of the five categories are given in Table 3.

**Table 3:** Mean values of fracture resistance in different categories N: Newton

Categories	Fracture resistance
Category 1	1208.87 N
Category 2	614.89 N
Category 3	733.05 N
Category 4	1179.14 N
Category 5	1148.49 N

The fracture resistance was maximum in Category 1 as it consisted of tooth specimens that were completely intact. The values of resistance against fracture in Category 1 were greater than that of Category 2 and Category 3 and the difference in findings of Category 1 compared to Category 2 and Category 3 was statistically significant. (p=0.002). The values of resistance against fracture in Category 1 were greater than that of Category 4 and Category 5, however, the difference in findings of Category 1 compared to Category 4 and Category 5 was statistically non-significant (p=0.671). On comparing Category 2 and Category 3, the values of fracture resistance were greater in Category 3 as compared to Category 2; however, the difference was non-significant statistically (p = 0.891). On comparing Category 2 with Category 4 and Category 5, the values of fracture resistance in Category 2 were lower than in Category 4 and Category 5 and the difference was significant statistically (p=0.001). On comparing Category 3 with Category 3 and Category 5, the values of fracture resistance in Category 3 were lower than in Category 4 and Category 5 and the difference was significant statistically (p=0.004). On comparing mean values of resistance against fracture in Category 4 and category 5, the the difference in values was non-significant statistically (p= 0.897) (Table 4).

**Table 4:** Intergroup comparisons of values of fracture resistance

Variable	P value
Category 1 versus Category 2 and Category 3	0.002
Category 1 versus Category 4 and Category 5	0.671
Category 2 versus Category 3	0.891
Category 2 versus Category 4 and Category 5	0.001
Category 3 versus Category 4 and Category 5	0.004
Category 4 versus Category 5	0.897

The values of resistance against fracture in intact tooth was greater than that in specimens where an inlay cavity preparation was done but not filled and specimens in which composite inlay restoration was prepared by the laboratory indirectly and the difference in findings was statistically significant.

The values of resistance against fracture in intact teeth were greater than that of tooth specimens with inlay formed of traditional monolithic ceramic derived from zirconia and tooth specimens with inlay formed of monolithic translucent ceramic derived from zirconia; however, the difference in findings was statistically non-significant.

 The values of fracture resistance were greater in composite inlay restoration prepared by the laboratory indirectly as compared to specimens where an inlay cavity preparation was done but not filled; however, the difference was non-significant statistically.

The values of fracture resistance in specimens where an inlay cavity preparation was done but not filled were lower than traditional monolithic ceramic derived from zirconia and tooth specimens with inlay formed of monolithic translucent ceramic derived from zirconia and the difference was significant statistically (p=0.001).

The values of fracture resistance in composite inlay restoration prepared by the laboratory indirectly were lower than monolithic ceramic derived from zirconia and tooth specimens with inlay formed of monolithic translucent ceramic derived from zirconia and the difference was significant statistically (p=0.004).

## Discussion

There is a dearth of information in the literature about how indirect zirconia-derived ceramic inlays and indirect laboratory-prepared composite restorations affect a tooth's resistance to fracture. The goal of the current study was to compare and evaluate the resistance to fracture of dental materials used in the indirect preparation of inlay restorations, such as composite restorations, monolithic translucent ceramic derived from zirconia, and traditional monolithic ceramic derived from zirconia.

The mean values of resistance against fracture in specimens in categories 1-5 were 1208.87 N, 614.89 N, 733.05 N, 1179.14 N, and 1148.49 N. The values of fracture resistance in composite inlay restorations prepared by laboratories indirectly were lower than those of monolithic ceramic derived from zirconia and tooth specimens with inlays formed of monolithic translucent ceramic derived from zirconia, and the difference was significant statistically (p=0.004). On comparing Category 3 with categories 4 and 5, the values of fracture resistance in Category 3 were found to be lower and the difference was significant statistically. (p=0.004).

This outcome was consistent with earlier research, which showed that restorations involving zirconia-based ceramic inlays may restore tooth structural rigidity more effectively than composite inlays [11-16]. The findings of our study were consistent with those of a prior investigation, which found that zirconia ceramic inlays placed on restored teeth had a fracture resistance comparable to that of unrestored teeth [17].

Numerous investigations have shown that samples repaired using indirect composite resin display a greater degree of fracture than samples repaired using ceramic material [15]. This behavior was also observed in the current study, where indirect composite-restored premolars had catastrophic fractures. In this study, the prototype was sprayed with an antireflection lacquer to produce an optical image. The model was scanned using an optical strip-light scanning system that was fully computerized and connected to a CAD/CAM workstation. After precisely drawing all cavity margins on the digital image, a particular version of software created for inlays was used to produce the framework. After being conceived, the physical prototype was created using VITA In-Ceram YZ cubes.

The values of resistance against fracture in intact teeth were greater than those of tooth specimens with inlays formed of traditional monolithic ceramic derived from zirconia and tooth specimens with inlays formed of monolithic translucent ceramic derived from zirconia; however, the difference in findings was statistically non-significant. The values of resistance against fracture in Category 1 were greater than those in categories 4 and 5; however, the difference was statistically non-significant (p=0.671).

The values of resistance against fracture in intact teeth were greater than those of specimens where an inlay cavity preparation was done but not filled and specimens where composite inlay restorations were prepared by a laboratory indirectly, and the difference in findings was statistically significant. The fracture resistance was maximum in Category 1, as it consisted of tooth specimens that were completely intact, and it was more than that in categories 2 and 3, and the difference in the findings in Category 1 compared to categories 2 and 3 was statistically significant (p=0.002).

The biomechanical and biocompatibility characteristics of the remaining dental structure, as well as aesthetic concerns, are related to the need for the restoration of teeth in the posterior region. One of the materials typically used for tooth-colored indirect inlay restorations on posterior teeth is zirconia [11,12]. Zirconia has some qualities, particularly in terms of utility, such as improved aesthetics, good physical strength, and greater mechanical strength. When selecting restorative materials for indirect inlay preparations for posterior teeth, it is important to analyze the many characteristics of each dental material, both functionally and aesthetically [13,14].

In this study, after being kept for a full week following the cementation of inlay replacements, the research samples underwent thermocycling. The samples were then subjected to axial compressive force by a metal globe applied vertically at a crosshead rate of 1 mm/minute in a universal testing device. The values of fracture resistance in specimens where an inlay cavity preparation was done but not filled were lower than those in traditional monolithic ceramic derived from zirconia and tooth specimens with inlays formed of monolithic translucent ceramic derived from zirconia, and the difference was significant statistically (p=0.001). On comparing Category 2 with categories 4 and 5, the values of fracture resistance in Category 2 were lower than those in categories 4 and 5, and the difference was significant statistically (p=0.001).

The nanocomposite used in this study uses cutting-edge, contemporary light dispersion technology. It has uniformly dispersed single-spread nanofillers that provide exceptional flexible durability and durability against wear, and it is globally sculptable [18,19]. An inlay, which is an intra-crown cast restoration, can be used to restore the occlusal surfaces and proximal surface of a posterior tooth without compromising the tooth's cusps. When an inlay is made using an indirect method, problems with conventional filling techniques, including poor occlusal or proximal aspect morphology, insufficient wear resistance, or inferior mechanical characteristics of directly inserted filler material, are resolved [20, 21].

Any pre-existing repair flaws that may appear throughout the production procedure and cyclic loading assessment could further encourage the formation of a fracture. This is why CAD/CAM technology was employed in the research. This prevents the formation of cracks by fabricating an indirect replacement in just one appointment. Zirconia's higher flexibility or fracture load resilience, which is around 2.5 times higher than pressable glass ceramic-based substances, was similarly observed in earlier investigations. Therefore, materials based on zirconia might be better suited for stress-bearing restorative areas [22,23].

The study has some limitations. In vitro procedures were used to conduct this investigation. As a result, values of resistance may change under clinical circumstances. Only vertically axial loads were used in this laboratory experiment; however, clinically, lateral pressure and fatigue load also have a significant effect. It would have been possible to evaluate the resistance to fracture of direct restoration components compared to that of indirect restorations.

## Conclusions

Within the constraints of the current investigation, we can state that indirect zirconia-based ceramic products offer adequate fracture resistance. This implies that these materials have the potential to withstand external forces without breaking, but additional research is needed to determine how well these materials hold up under different types of pressures before employing them in clinical tooth restoration. This could include analyzing their response to biting forces, chewing, or other forms of mechanical stress that teeth may experience during everyday use.
